# Characterization of a caprine model for the subclinical initial phase of *Mycobacterium avium* subsp. *paratuberculosis* infection

**DOI:** 10.1186/s12917-015-0381-1

**Published:** 2015-03-24

**Authors:** Heike Köhler, Anneka Soschinka, Michaela Meyer, Angela Kather, Petra Reinhold, Elisabeth Liebler-Tenorio

**Affiliations:** Institute of Molecular Pathogenesis, Friedrich-Loeffler-Institut, Federal Research Institute for Animal Health, Jena, Germany; Present address: Tierärztliche GmbH Hagenow, Hagenow, Germany; Present address: Tierarztpraxis Dr. Peitzmeier, Hille, Germany; Present address: Institute of Immunology, Jena University Hospital, Jena, Germany

**Keywords:** Experimental animal model, MAP, Goat, Faecal shedding, Antibody, IFN-γ, Culture, Pathology

## Abstract

**Background:**

Paratuberculosis caused by *Mycobacterium avium* subsp. *paratuberculosis* (MAP) is difficult to control due to a long phase of clinically non-apparent (latent) infection for which sensitive diagnostics are lacking. A defined animal model for this phase of the infection can help to investigate host-MAP interactions in apparently healthy animals and identify surrogate markers for disease progress and might also serve as challenge model for vaccines. To establish such a model in goats, different age at inoculation and doses of oral inoculum of MAP were compared. Clinical signs, faecal shedding as well as MAP-specific antibody, IFN-γ and IL-10 responses were used for in vivo monitoring. At necropsy, about one year after inoculation (pi), pathomorphological findings and bacterial organ burden (BOB) were scored.

**Results:**

MAP infection manifested in 26/27 inoculated animals irrespective of age at inoculation and dose. Clinical signs developed in three goats. Faecal shedding, IFN-γ and antibody responses emerged 6, 10–14 and 14 wpi, respectively, and continued with large inter-individual variation. One year pi, lesions were detected in 26 and MAP was cultured from tissues of 23 goats. Positive animals subdivided in those with high and low overall BOB. Intestinal findings resembled paucibacillary lesions in 23 and multibacillary in 4 goats. Caseous and calcified granulomas predominated in intestinal LNN. BOB and lesion score corresponded well in intestinal mucosa and oGALT but not in intestinal LNN.

**Conclusions:**

A defined experimental infection model for the clinically non-apparent phase of paratuberculosis was established in goats as suitable basis for future studies.

## Background

Paratuberculosis is a chronic granulomatous enteritis caused by *Mycobacterium avium* subsp. *paratuberculosis* (MAP) and affects domestic and wild ruminants worldwide, causing considerable economic losses for the livestock industry [1,2]. Research to improve diagnostic methods and prophylactic measures has been performed for many years, but still many questions remain unanswered. One reason is the extended clinically non-apparent initial phase of the infection and the still insufficient knowledge about the interactions between the host organism and the pathogen during this time period.

Despite large numbers of naturally MAP infected animals, elucidation of host-pathogen interactions in the early phase of the disease is only possible using the defined conditions and variables of experimental animal models. This is due to a diagnostic gap that allows in vivo identification of infected animals only after sero-conversion or after the onset of faecal shedding, which become detectable late in the course of the disease with large inter-individual variation [3]. Experimental animal infection models allow the investigation of relevant numbers of animals with defined infection status and under identical conditions during the clinically non-apparent phase of disease.

Experimental infections have been performed in diverse domestic species, and furthermore, in small laboratory animals [4]. Study conditions were not standardized among the experiments making comparisons difficult. Generally, age at infection, dose and frequency of inoculation, and duration of the experiment are decisive for disease development [4,5]. International guidelines for standardization of animal models for paratuberculosis have been proposed only recently [5]. While cattle, sheep and deer have been used extensively studies in goats are rare and only small numbers of animals were included [6-12]. Since marked individual variations of host immune response and lesions were observed even in the same experiment, the conclusions vary widely.

Performing experimental infections in goats has several advantages in comparison to cattle and sheep. Goats are susceptible to the three main groups of MAP, Type I, II and III [13-15]. They are considered the least naturally MAP resistant species due to a rather fast disease progress [16]. This allows a shorter duration of experiments. In a study using Angora goats, specific IFN-γ responses were observed already one month after challenge with MAP positive gut mucosa and sero-conversion as early as four months post infection (mpi). Clinical signs occurred between 22 and 29 mpi [17]. In addition, the feeding and housing requirements of goats are easier to fulfil compared to cattle.

The aim of the present study was to establish a well characterized experimental animal model for the clinically non-apparent phase of paratuberculosis in goats as a basis for future studies of the early pathogenesis of MAP infection.

## Results

### Clinical signs

Severe clinical signs of paratuberculosis were observed in three of the MAP-inoculated animals (3/27). One animal each of group V2 and V4 developed non-treatable diarrhea at 37 and 35 wpi, respectively, and had to be necropsied, while the third goat of group V1 was cachectic at 48 wpi. At 37, 38 and 39 wpi, 3 other goats of group V2 died or had to be euthanized because of neurologic signs. Post mortem examination revealed cerebrocortical necrosis.

### Shedding of MAP

MAP was detected repeatedly in the faeces of most of the animals during the inoculation period (not shown). Shedding stopped at 1 wpi in 13 of the 14 early inoculated goats and in eight of the 13 late inoculated goats and re-emerged about 6 wpi in all animals except one goat of group V2. A large inter-individual variability of shedding in terms of intensity (not shown) and time course was observed independent from inoculation time and dose. Essentially, three different shedding patterns occurred: animals that stopped shedding before 34 wpi (1), animals that shed MAP intermittently until necropsy (2) and animals that shed MAP continuously during the entire course of the experiment (3). Animals which had to be necropsied before the end of the observation period were not defined (Table 1).

**Table 1:** **Faecal shedding of MAP before and after oral inoculation and shedding category of animal**

**Group**	**Animal No.**	**Week p. i.**														**Shedding category** ^**a)**^
		**-3**	**1**	**6**	**10**	**14**	**18**	**22**	**26**	**30**	**34**	**38**	**42**	**44**	**46**	
**K1**	**1**	-	-	-	-	-	-	-	-	-	-	-	-	-	-	**-**
	**2**	-	-	co.	-	-	-	-	-	-	-	-	#			**-**
	**3**	-	n.a.	-	-	-	-	-	-	-	-	-	-	-	-	**-**
	**4**	-	-	co.	-	-	-	-	-	-	-	-	-	-	-	**-**
	**5**	-	-	co.	-	-	-	-	-	-	-	-	-	-	-	**-**
	**6**	-	-	-	-	-	-	-	-	-	-	-	-	-	-	**-**
**V1**	**7**	-	-	**X**	**X**	**X**	**X**	**X**	**X**	-	-	-	-	-	-	**1**
	**8**	-	-	**X**	**X**	**X**	**X**	**X**	**X**	**X**	**X**	**X**	**X**	**X**	**X**	**3**
	**9**	-	-	**X**	**X**	**X**	**X**	**X**	**X**	**X**	**X**	**X**	**X**	**X**	**X**	**3**
	**10**	-	-	**X**	**X**	**X**	**X**	**X**	**X**	**X**	**X**	**X**	**X**	**X**	**X**	**3**
	**11**	-	-	**X**	**X**	**X**	**X**	**X**	**X**	**X**	-	-	-	-	-	**1**
	**12**	-	-	**X**	**X**	**X**	**X**	**X**	**X**	**X**	-	-	-	-	-	**1**
	**13**	-	-	**X**	**X**	**X**	-	-	-	-	-	**X**	**X**	**X**	**X**	**2**
**V2**	**14**	-	-	**X**	**X**	**X**	**X**	**X**	**X**	-	**X**	-	#			**nd**
	**15**	-	-	**X**	**X**	-	-	**X**	-	-	-	-	**X**	-	-	**2**
	**16**	-	-	**X**	**X**	**X**	**X**	**X**	**X**	**X**	-	#				**nd**
	**17**	-	**X**	**X**	**X**	**X**	**X**	**X**	-	-	-	-	#			**nd**
	**18**	-	-	**X**	**X**	**X**	**X**	**X**	**X**	-	-	-	-	-	-	**1**
	**19**	-	-	-	**X**	**X**	**X**	**X**	**X**	**X**	**X**	**X**	**X**	**X**	**X**	**3**
	**20**	-	-	**X**	**X**	-	-	-	n.a.	-	-	-	#			**nd**
**K2**	**21**	-	-	-	-	-	-	-	-	-	-	-	-	-	-	**-**
	**22**	-	-	-	-	-	-	-	-	-	-	-	-	-	-	**-**
	**23**	-	-	-	-	-	-	-	-	-	-	-	-	-	-	**-**
	**24**	-	-	-	-	-	-	-	-	-	-	-	-	-	-	**-**
	**25**	-	-	-	-	-	-	-	-	-	-	-	-	-	-	**-**
	**26**	-	-	-	-	-	-	-	-	-	-	-	-	-	-	**-**
**V3**	**27**	-	**X**	**X**	**X**	**X**	**X**	-	-	**X**	**X**	**X**	**X**	**X**	**X**	**2**
	**28**	-	-	**X**	**X**	**X**	**X**	**X**	**X**	**X**	-	-	-	-	-	**1**
	**29**	-	**X**	**X**	**X**	**X**	**X**	**X**	-	-	-	-	-	-	#	**nd**
	**30**	-	-	**X**	**X**	**X**	**X**	**X**	-	-	-	-	-	**X**	-	**2**
	**31**	-	-	**X**	**X**	**X**	-	-	-	-	-	-	-	-	-	**1**
	**32**	-	**X**	**X**	**X**	**X**	-	**X**	-	-	-	-	-	-	-	**1**
**V4**	**33**	-	-	**X**	**X**	**X**	**X**	**X**	**X**	-	-	**X**	-	**X**	-	**2**
	**34**	-	-	**X**	**X**	-	-	-	-	-	-	-	**X**	-	-	**2**
	**35**	-	**X**	**X**	**X**	**X**	**X**	**X**	**X**	**X**	**X**	**X**	**X**	**X**	**X**	**3**
	**36**	-	-	**X**	**X**	**X**	-	-	-	-	**X**	#				**nd**
	**37**	-	-	**X**	**X**	**X**	**X**	**X**	**X**	**X**	**X**	**X**	**X**	**X**	**X**	**3**
	**38**	-	-	**X**	**X**	**X**	**X**	-	-	-	-	-	-	-	-	**1**
	**39**	-	**X**	**X**	**X**	-	-	-	-	-	-	-	-	-	-	**1**

^a)^Shedding category: 1 – stopping before 34 wpi; 2 – intermittent; 3 – continuously; **X**, positive faecal culture; n.a., not available; co., contaminated; #, animal no longer available (necropsy); nd, not defined.

### Antibody response

**Figure 1:**
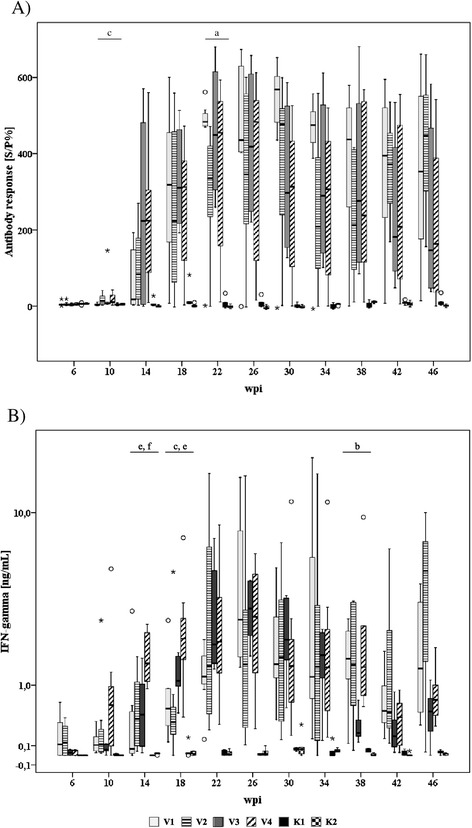
**Time course and intensity of MAP-specific antibody response (A) and antigen-induced (Johnin, 4 μg/mL) IFN-γ response (B) of inoculated and control goats.** Box and Whisker Plot represents median value, 25% and 75% percentiles (box), range, outlier values (○), and extreme values (*). Different letters indicate significant differences between groups (Mann–Whitney-U test, P ≤ 0.05): a – V1 vs. V2, b – V1 vs. V3, c – V1 vs. V4, d – V2 vs. V3, e – V2 vs. V4, f – V3 vs. V4.

The specific antibody response against MAP started at 14 wpi in the MAP-inoculated animals. The proportion of antibody positive animals as well as antibody levels increased until 22–26 wpi. S/P% varied largely between individuals with no significant differences between inoculation groups in general (Figure 1A). No seroconversion was observed in three MAP-inoculated goats from different groups (V1, V2 and V4) and in all control animals.

### IFN-γ response

A specific IFN-γ response of the MAP-inoculated animals against jPPD was first detected at 10–14 wpi. IFN-γ release of the PBMC reached peak values at 22–26 wpi and decreased afterwards. At 14 and 18 wpi, the IFN-γ response of group V4 (late inoculated animals that received 20 mg bwm per dose) exceeded that of the other inoculated groups. Because of the large variation of the response within the groups, statistically significant differences between inoculation groups occurred only incidentally. In the control groups no jPPD-specific IFN-γ release was induced (Figure 1B).

### IL-10 response

**Figure 2:**
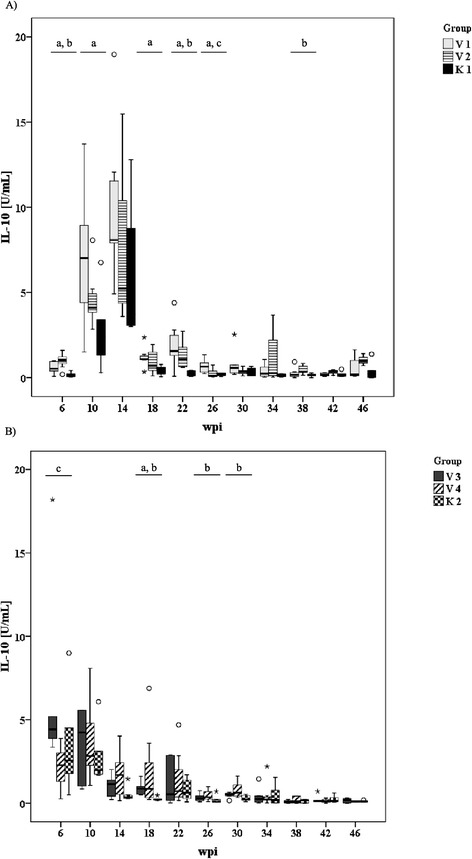
**Time course and intensity of the antigen-induced (Johnin, 4 μg/mL) IL-10 response of early inoculated goats (A) and late inoculated goats (B) and the corresponding control animals.** Box and Whisker Plot: see Figure 1. Different letters indicate significant differences between groups (Mann-Whitney-U test, P ≤ 0.05): Figure 2A: a – K1 vs. V1, b – K1 vs. V2, c – V1 vs. V2; Figure 2B: a – K2 vs. V3, b – K2 vs. V4, c – V3 vs. V4.

A marked release of IL-10 was induced by in vitro stimulation of PBMC from MAP-inoculated and control animals with jPPD at 6–14 wpi (K1, V1 and V2, Figure 2A) or 6–10 wpi (K2, V3 and V4, Figure 2B). Time course and magnitude of the response differed between early and late inoculated groups including the respective controls with peak responses at 14 wpi in group K1, V1 and V2 and at 10 wpi in groups K2, V3 and V4. The control groups tended to have a lower IL-10 response than the two age matched MAP-inoculated groups. This was more pronounced in the younger animals (K1, V1 and V2). At later sampling dates the IL-10 response of all groups decreased considerably (Figure 2A and B).

### Bacterial organ burden (BOB)

MAP was culturally isolated after necropsy from at least one tissue sample of 23 of the 27 MAP-inoculated goats. The four MAP negative goats belonged to the groups V1 (n = 2), V2 (n = 1) and V4 (n = 1). These animals were allocated to BOB category I. MAP positive animals could be sub-divided into two categories (Table 2):

**Table 2:** **Cultural detection of MAP in intestinal mucosa, oGALT and intestinal lymph nodes of the MAP-inoculated goats at necropsy**

**Group**	**Animal No.**	**Jejunal LNN**			**Ileocol**	**LNN**		**JPP**	**IPP**		**ICVPP**	**PCPP**	**oGALT**		**Duoden**	**Jejunum**			**Intestine**		**Summary BOB cat.**
		**prox.**	**median**	**distal**	**LN**	**BOB**	**BOB cat.**		**prox.**	**distal**			**BOB**	**BOB cat.**		**1**	**2**	**3**	**BOB**	**BOB cat.**	
**V1**	7	0^a)^	0	0	0	**0**	**I** ^**b)**^	0	0	0	0	0	**0**	**I**	0	0	0	0	**0**	**I**	**I**
	8^c)^	3.85	25.00	25.00	37.50	**22.84**	**III**	25.00	0	20.14	21.53	21.53	**17.64**	**III**	25.00	8.42	8.33	20.14	**15.47**	**III**	**III**
	9	22.63	50.00	40.00	40.00	**38.16**	**III**	40.00	35.00	40.00	62.50	co.	**44.38**	**III**	co.	23.57	40.00	40.00	**34.52**	**III**	**III**
	10	15.00	32.50	25.00	40.00	**28.13**	**III**	38.75	33.33	26.25	co.	co.	**32.78**	**III**	7.50	40.00	38.75	37.50	**30.94**	**III**	**III**
	11	0	4.96	6.75	6.25	**4.49**	**II**	0	0	0	3.13	0	**0.63**	**II**	co.	0	16.52	0	**5.51**	**II**	**II**
	12	0	0	0	0	**0**	**I**	0	0	0	co.	0	**0**	**I**	0	0	0	0	**0**	**I**	**I**
	13	17.80	11.11	0	22.22	**12.78**	**II**	0	0	0	22.22	5.56	**5.55**	**II**	0	16.67	16.67	25.00	**14.58**	**II**	**II**
**V2**	14^d)^	0	0	7.29	0	**1.82**	**II**	0	0	co.	8.33	0	**2.08**	**II**	0	0	co.	co.	**0**	**I**	**II**
	15	16.67	8.33	8.33	8.33	**10.42**	**II**	16.67	0	4.17	0	8.33	**5.83**	**II**	0	8.33	0	0	**2.08**	**II**	**II**
	16^e)^	12.46	8.33	10.06	0	**7.71**	**II**	14.38	16.67	12.79	12.11	0	**11.19**	**II**	0	12.84	0	5.55	**4.60**	**II**	**II**
	17^f)^	0	0	0	7.50	**1.88**	**II**	n.a.	0	0	0	1.85	**0.46**	**II**	0	0	0	0	**0**	**I**	**II**
	18	0	0	0	0	**0**	**I**	0	0	0	0	0	**0**	**I**	0	0	0	0	**0**	**I**	**I**
	19	6.25	20.83	25.00	31.89	**20.97**	**III**	20.83	11.11	26.39	16.67	co.	**18.75**	**III**	0	16.67	20.83	20.83	**14.58**	**II**	**III**
	20^g)^	0	0	0	0	**0**	**I**	0	0	0	0	0	**0**	**I**	0	16.67	0	0	**0.42**	**II**	**II**
**V3**	27	17.14	12.33	15.48	36.61	**20.39**	**III**	4.17	9.72	15.47	16.67	8.33	**10.87**	**II**	0	15.08	32.14	20.24	**16.87**	**III**	**III**
	28	13.26	14.44	4.17	9.33	**10.30**	**II**	8.33	2.17	0	9.33	co.	**4.96**	**II**	5.00	8.33	11.11	10.00	**8.61**	**II**	**II**
	29	0	4.80	5.88	0	**2.67**	**II**	co.	0	0	5.00	co.	**1.67**	**II**	0	0	0	0	**0**	**I**	**II**
	30	0	3.75	0	8.69	**3.11**	**II**	16.67	0	2.50	10.00	33.33	**12.50**	**II**	0	10.00	0	0	**2.50**	**II**	**II**
	31	0	0	0	8.63	**2.16**	**II**	co.	0	0	0	0	**0**	**I**	co.	0	0	0	**0**	**I**	**II**
	32	0	0	0	2.00	**0.5**	**II**	0	0	0	0	co.	**0**	**I**	0	0	0	0	**0**	**I**	**II**
**V4**	33	10.24	12.54	9.42	20.51	**13.18**	**II**	4.76	0	0	0	18.89	**4.73**	**II**	0	8.33	25.00	0	**8.33**	**II**	**II**
	34	9.40	16.91	0	11.11	**9.35**	**II**	co.	0	0	0	0	**0**	**I**	0	0	19.09	8.33	**6.86**	**II**	**II**
	35	33.33	33.33	1.85	41.67	**27.55**	**III**	37.37	0	25.00	16.67	20.21	**19.85**	**III**	4.17	16.67	25.00	16.67	**15.62**	**III**	**III**
	36^h)^	8.89	13.29	16.67	0	**9.71**	**II**	0	10.00	8.33	14.34	13.33	**9.20**	**II**	3.33	0	co.	0	**1.11**	**II**	**II**
	37	35.00	30.00	35.00	40.00	**35.00**	**III**	33.33	23.81	8.33	co.	4.76	**17.56**	**III**	0	33.33	35.71	10.00	**19.76**	**III**	**III**
	38	6.67	0	18.75	15.86	**10.31**	**II**	co.	co.	co.	0	0	**0**	**I**	0	co.	co.	0	**0**	**I**	**II**
	39	0	0	0	0	**0**	**I**	0	0	0	0	0	**0**	**I**	0	0	0	0	**0**	**I**	**I**

^a)^Growth Index ^b)^BOB category: I, no growth; II, BOB ≤ 15; III, BOB > 15; ^c)^cachectic; ^d)^died 39 wpi; ^e)^necropsied 37 wpi, diarrhea; ^f)^died 37 wpi; ^g)^necropsied 38 wpi; ^h)^necropsied 35 wpi, diarrhea; prox. proximal; Ileocol Ileocolic; Duoden Duodenum; co. contaminated; n.a. not available.

BOB category II: Animals that harbored only low to moderate amounts of MAP in intestinal tissues and associated lymph nodes. The proportion of MAP positive organs varied largely between these animals. MAP could be recovered most often from the ICV-LN, followed by the M-LN and ICVPP. The IPP and the mucosa of the duodenum were positive only in a few cases.

BOB category III: Animals that had moderate to very high amounts of MAP in most of the samples from intestinal tissues and associated lymph nodes. High amounts of MAP were most frequently recovered from the ICV-LN, the proximal and median jejunal LN and the mucosa of the mid jejunum.

MAP was also isolated from extra-intestinal tissue of nine animals, three from group V1, two each from groups V2 and V4 and one from group V3. Regarding lymphatic tissue, the hepatic LN was most often positive (n = 8), followed by the retropharyngeal LN (n = 5) and the tonsils (n = 2). MAP was isolated from liver of five and spleen of two animals (Table 3). Notably, most tissue samples of the cachectic goat were MAP positive, including superficial cervical LN, kidney, diaphragm and gluteal muscle (not shown).

**Table 3:** **Cultural isolation of MAP and lesions in extra-intestinal sites**

**Group**	**Animal No.**	**Liver**			**Hepatic LN**			**Spleen**			**Retropharyngeal LN**			**Tonsil**		
		**Culture**	**gInf**	**MAP-IHC**	**Culture**	**gInf**	**MAP-IHC**	**Culture**	**gInf**	**MAP-IHC**	**Culture**	**gInf**	**MAP-IHC**	**Culture**	**gInf**	**MAP-IHC**
**V1**	7	0^a)^	0	0	0	0	0	0	0	0	0	0	0	0	0	0
	8	33.33	+++^b)^	0	25.00	++	0	12.50	0	0	10.42	0	0	26.04	++	0
	9	0	0	0	13.93	0	0	0	0	0	0	0	0	0	0	0
	10	21.78	++	0	14.16	0	0	11.46	0	0	0	0	0	0	0	0
	11	0	0	0	0	0	0	0	0	0	0	0	0	0	0	0
	12	0	+	0	0	0	0	0	0	0	0	0	0	0	0	0
	13	0	0	0	0	0	0	0	0	0	0	0	0	0	0	0
**V2**	14^c)^	co.	0	0	0	++	0	0	0	0	0	0	0	0	0	0
	15	0	0	0	0	0	0	0	0	0	0	0	0	0	0	0
	16^c)^	0	0	0	1.9	+	0	0	0	0	0	0	0	0	0	0
	17^c)^	0	0	0	0	++	0	0	0	0	5.26	0	0	0	0	0
	18	0	0	0	0	0	0	0	0	0	0	0	0	0	0	0
	19	0	+++	0	6.6	++	0	0	0	0	4.16	+	0	3.57	0	0
	20^c)^	0	0	0	0	0	0	0	0	0	0	0	0	0	0	0
**V3**	27	12.50	0	0	8.33	0	0	0	0	0	0	0	0	0	0	0
	28	co.	0	0	0	0	0	0	0	0	0	0	0	0	0	0
	29	0	0	0	0	0	0	0	0	0	0	0	0	0	0	0
	30	0	0	0	0	0	0	0	0	0	0	0	0	0	0	0
	31	0	0	0	0	0	0	0	0	0	0	0	0	0	0	0
	32	0	0	0	0	0	0	0	0	0	0	0	0	0	0	0
**V4**	33	0	0	0	0	0	0	0	0	0	0	0	0	0	0	0
	34	0	0	0	0	0	0	0	0	0	0	0	0	0	0	0
	35	16.78	0	0	8.33	0	0	0	0	0	8.33	0	0	0	0	0
	36^d)^	0	0	0	0	0	0	0	0	0	0	0	0	0	0	0
	37	4.29	0	0	22.50	0	0	0	0	0	7.94	0	0	0	0	0
	38	co.	0	0	0	0	0	0	0	0	0	0	0	0	0	0
	39	co.	0	0	0	0	0	0	0	0	0	0	0	0	0	0

^a)^Growth Index ^b)^granulomatous infiltrate: 0, none; +, mild, focal; ++, moderate, multifocal; +++, severe, diffuse; ^c)^necropsy at 37–39 wpi; ^d)^necropsy at 35 wpi; co. contaminated; na. not available; gInf, granulomatous infiltrate; MAP IHC, MAP antigen detected by IHC.

On the individual animal level, no marked differences in the BOB of intestinal lymph nodes, oGALT and intestinal mucosa could be detected in animals with BOB category III, while variability was higher in category II animals. Culturally negative and the two categories of culturally positive animals were distributed over all inoculation groups.

### Gross pathology

**Figure 3:**
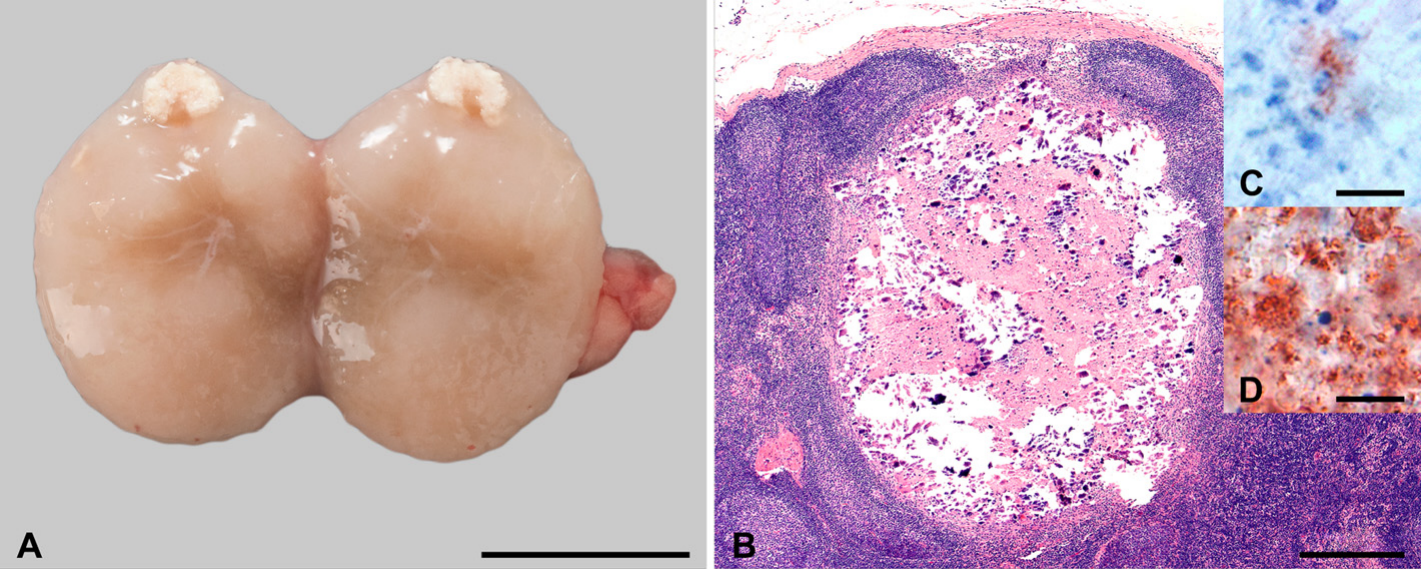
**Lesions in mesenteric lymph nodes.** Enlarged lymph node with focal necrosis and calcification (**A**, goat 9, bar = 1 cm), granuloma with extensive necrosis and calcification (**B**, goat 38, HE, bar = 500 μm). Small foci of granular staining for mycobacteria possibly associated with a degenerated cell were seen in granulomas of most goats (**C**, goat 11, immunohistochemistry MAP, bar = 10 μm), numerous mycobacteria throughout granulomas in 4 goats (**D**, goat 19, immunohistochemistry MAP, bar = 10 μm).

**Figure 4:**
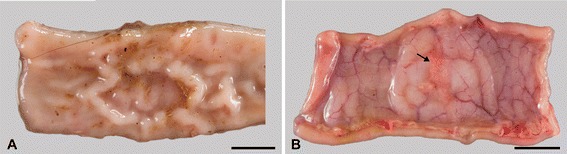
**Lesions at Peyer´s patches.** Indented Peyer’s patch in jejunum. (**A**, goat 13). Circumscribed villous serositis (arrow) is frequent in altered Peyer’s patches (**B**, goat 31). Bar = 1 cm.

Macroscopic lesions were most frequently (21 out of 27 goats) seen in mesenteric and ileocolic lymph nodes and oGALT, especially JPPs. Affected lymph nodes were enlarged and had areas of necrosis and calcification varying from 1 mm to extensive throughout the entire lymph node (Figure 3A). JPPs had reduced thickness and were indented in most of the goats (Figure 4A), in a few goats they were thickened and firm (Table 4). The surface was occasionally ulcerated. Chronic villous serositis was regularly seen at the serosal aspect of altered JPPs (Figure 4B). Lesions were inconsistently seen in the IPP, ICVPP and PCPP. Both intestinal lymph nodes as well as oGALT were altered in most goats. Intestinal wall outside oGALT was affected less frequently (12 out of 27 goats, Table 4). Multiple small (0.5–2 cm) foci of thickened intestinal mucosa were seen in the small intestine of seven, thickening of segments in three and of the entire length in another three goats. The latter was associated with corrugated intestinal mucosa. Thickened and nodular lymphatics were frequent in the altered segments of intestine. Intestinal lesions were restricted to the small intestine and were seen only in goats which had also lesions in JPPs and intestinal lymph nodes. All goats from the control groups and one goat of groups V3 and V4 each were without paratuberculous lesions. A few helminths were seen in a few goats of all groups.

**Table 4:** **Lesions in intestinal lymph nodes, oGALT and small intestine of the MAP-inoculated goats at necropsy: macroscopic and histological lesions and MAP detection by IHC**

**Group**	**Anim. No.**	**Mesenteric and ileocolic LNN**				**oGALT (e.g. JPP, #of sites examined = 7)**					**Small intestine (#of sites examined = 5)**					**Lesion cat. summary**
		**Macro**	**Histo**	**MAP IHC**	**Lesion cat. LNN**	**Macro**	**Histo**	**MAP IHC**	**#of sites affected**	**Lesion cat. oGALT**	**Macro**	**Histo**	**MAP IHC**	**#of sites affected**	**Lesion cat. intestine**	
**V1**	7	✓, e	G	2	III	-	-	0	0	I	-	-	0	0	I	III
	8	✓, e	G, gInf	2	III	✓	mf , D	2	7	III	✓, E	mf +++	2	4	III	III
	9	✓, e	G, gInf	1	II	✓	mf , C	2	5	III	✓, S	mf ++	0	4	III	III
	10	✓, f	G, gInf	1	III	✓	mf, C	1	4	III	✓, S	mf +	2	4	III	III
	11	✓, e	G, gInf	1	III	✓	f/mf, C/A	0	2	II	-	f +	0	1	II	II
	12	✓, e	G	1	III	-	-	0	0	I	-	-	0	0	I	III
	13	-	gInf	1	II	✓	f/mf, B	0	1	II	✓, MF	mf +	0	2	II	II
**V2**	14^a^	✓, e	G, gInf	1	III	✓	f, C	0	3	II	-	-	0	0	I	II
	15	-	-	0	I	✓	mf, C	0	1	II	✓, MF	-	0	0	I	II
	16^a^	✓, e	G, gInf	1	III	✓	mf, B	0	3	II	✓, E	mf ++	0	4	III	III
	17^a^	✓, e	G, gInf	1	III	✓	mf, C	0	1	II	-	-	0	0	I	II
	18	✓, e	G, gInf	2	III	✓	mf, C	0	3	II	-	-	0	0	I	II
	19	✓, e	G, gInf	2	III	✓	mf/d, C	0	6	III	✓, S	mf ++	0	4	III	III
	20^a^	✓, f	G, gInf	1	II	-	-	0	0	I	-	-	0	0	I	II
**V3**	27	✓, f	G	0	II	✓	mf, C/A	0	5	III	✓, S, MF	f +	0	1	II	II
	28	✓, e	G, gInf	1	II	✓	mf, C	0	1	II	✓, MF	f ++	0	3	III	II
	29	✓, f	G, gInf	1	II	✓	f, C	0	2	II	-	-	0	0	I	II
	30	✓, f	G, gInf	1	II	✓	mf, C	0	4	III	-	-	0	0	I	II
	31	-	gInf	0	II	-	-	0	0	I	-	-	0	0	I	II
	32	✓, e	G, gInf	1	III	✓	f, C	0	2	II	-	f ++	0	1	II	II
**V4**	33	✓, e	G, gInf	1	III	✓	f/mf, C/A	0	2	II	✓, MF	mf +	0	1	II	II
	34	-	-	0	I	✓	mf, C	0	1	II	-	f +	0	2	II	II
	35	✓, e	G, gInf	1	III	✓	mf, C	0	7	III	✓, MF	mf ++	0	4	III	III
	36^b^	-	-	0	I	✓	mf, C	0	4	III	-	f +	0	1	II	II
	37	✓, e	G, gInf	1	III	✓	mf, D	2	4	III	✓, MF	mf +/++	1	4	III	III
	38	✓, e	G, gInf	1	III	-	-	0	0	I	-	-	0	0	I	III
	39	-	-	0	I	-	-	0	0	I	-	-	0	0	I	I

✓ paratuberculous lesion; − no paratuberculous lesion; LNN lymph nodes; G granuloma; gInf granulomatous infiltrate; MAP IHC MAP detected by IHC; nd not done; ^a^necropsy at 37–39 wpi; ^b^necropsy at 35 wpi; 0 no MAP; 1 paucibacillary; 2 multibacillary; f focal; mf multifocal; e extensive; A-D type of histological lesion (see text); +, ++, +++ mild, moderate, severe granulomatous infiltrates; E entire small intestine with thickened intestinal wall and corrugated mucosa; S segments of small intestine with thickened intestinal wall and corrugated mucosa; MF 0.5 – 2 cm thickening of the intestinal mucosa with central depression; lesion categories: I - no lesions; II – mild lesions; III - severe lesions.

### Histopathology and immunohistochemistry for MAP

Histopathology confirmed the macroscopic lesions in intestinal lymph nodes (Table 4). Granulomas with extensive central necrosis and calcification surrounded by a variable amount of granulomatous infiltrate, fibrocytes and lymphocytes predominated (Figure 3B). Small amounts of mycobacterial material (paucibacillary) were regularly seen by IHC in the necrotic centers (Figure 3C). Multibacillary lesions were seen in four of the early inoculated goats (Figure 3D). Multifocal granulomatous infiltrates of epitheloid cells and multinucleated giant cells (MGCs) were seen in subcapsular sinuses, along trabeculae and adjacent to granulomas in 18 goats additionally to granulomas and in 2 goats as only lesions. Single mycobacteria were detected occasionally in these infiltrates.

To allow better comparison with immunologic and bacterial culture data, lesions in intestinal lymph nodes were scored as no lesions (category I), mild lesions (category II) or severe lesions (category III). Animals with category III lesions predominated. Goats with mild lesions were particularly frequent in V3. One goat of V2 and three of V4 had no lesions in intestinal lymph nodes (category I).

**Figure 5:**
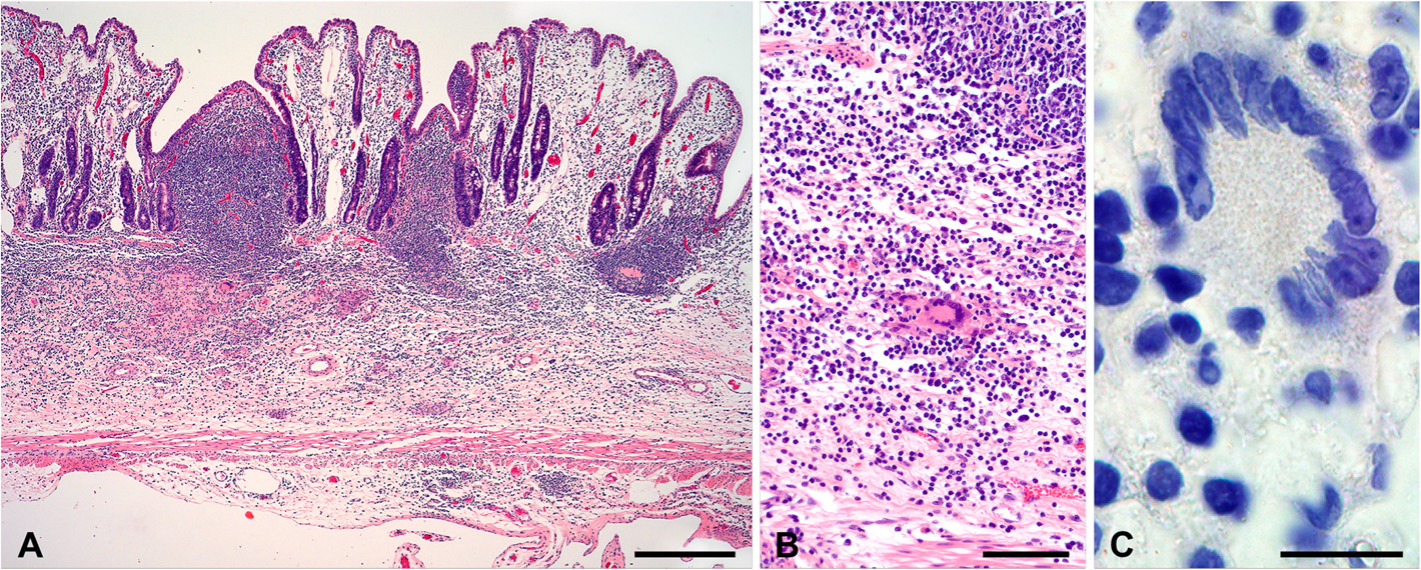
**Severely atrophic Peyer’s patch in jejunum lacking lymphoid follicles and interfollicular areas, but with marked villous serositis.** (**A**, goat 28). The organized lymphoid tissue is replaced by multiple small foci of epitheloid cells and MGCs and an extensive infiltrate of lymphocytes and plasma cells (**A**, **B** higher magnification of **A**, HE). There are no mycobacteria in the granulomatous infiltrate (**C**, immunohistochemistry MAP). Bar in A = 500 μm, bar in B = 100 μm, bar in C = 10 μm.

**Figure 6:**
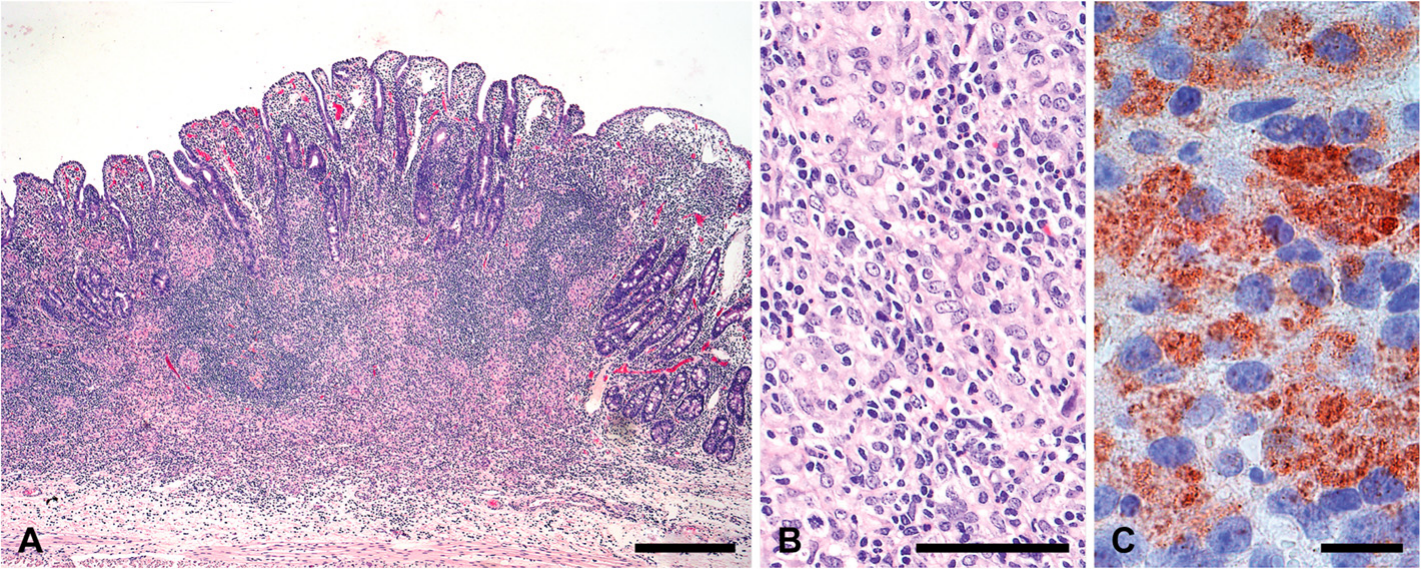
**Jejunum Peyer’s patch with severe granulomatous infiltrate.** (goat 37, **A**, **B** higher magnification of A, HE). Many mycobacteria in the granulomatous infiltrate (**C**, immunohistochemistry MAP). Bar in A = 500 μm, bar in B = 100 μm, bar in C = 10 μm.

Macroscopic lesions in JPPs were confirmed in all affected goats by histology. In the majority of goats (16 out of 20), the architecture of the organized lymphoid tissue was markedly altered with few and small lymphoid follicles and severe and sometimes complete loss of interfollicular lymphoid tissue (Figure 5A). Lesions were characterized by small focal to multifocal groups of epitheloid cells and few MGCs and extensive infiltration with lymphocytes and plasma cells (lesion type C, Figure 5B). The mucosal surface was irregular with occasional ulcers. A mild to severe chronic fibro-proliferative serositis was seen regularly. Lesions were paucibacillary in one goat, multibacillary in one goat and without mycobacteria by IHC in 14 goats (Figure 5C). In three goats, small granulomatous infiltrates without changes of the tissue architecture were seen in addition (lesion type A). Two goats from the early inoculated groups had more extensive infiltrates of epitheloid cells and MGCs admixed with lymphocytes predominantly in the interfollicular areas (lesion type B). Mycobacteria were not detected in type A and B lesions by IHC. Multifocal, moderate to severe infiltrates of predominantly epitheloid cells (lesion type D) occurred in one goat of group V1 and one goat of group V4 (Figure 6A, B). Numerous mycobacteria were present in the epitheloid cells of these lesions (Figure 6C). Comparable lesions were detected in other sites with oGALT, e.g. IPP, ICVPP, PCPP and in the rectum, but less frequently.

Lesions in the intestine outside oGALT were characterized by multifocal infiltrates of epitheloid cells and few MGCs. MGCs predominated in small and focal lesions which were often missed by the macroscopic examination. Mycobacteria were detected in 3 of the 15 goats with intestinal lesions.

Lesions in oGALT and small intestine were also classified as lesions category I to III. Besides the extent of lesions and number of mycobacteria detected by IHC, the number of oGALT (n = 7) and intestinal sites (n = 5) collected for histology with lesions was included. A mix of goats with all lesions categories were seen in all groups. Goats with lesions category II in oGALT and without lesions (category I) in small intestine were more frequent in group V2.

### Association between lesion category and BOB category

A clear association between lesion and BOB category was noted in oGALT and small intestinal tissue (Table 5, A and B). No to mild lesions predominated in those tissues where no bacterial growth had been detected. Tissues with bacterial organ burden were characterized by mild to severe lesions, the strongest association between severe lesions and high BOB was noted in the oGALT. In contrast, no association between lesion and BOB category could be established in the intestinal lymph nodes. Severe lesions predominated in LNN with low and even without BOB (Table 5, C).

**Table 5:** **Relation between histological score and bacterial organ burden of selected organs**

		**Bacterial organ burden category (n)**				**p χ** ^**2**^ **-test (Pearson)**
	**A) Intestinal mucosa**	**I**	**II**	**III**	**Total**	
**Lesion category (n)**	**I**	9	3	0	12	0.002
	**II**	1	5	1	7	
	**III**	0	4	4	8	
	**Total**	10	12	5	27	
	**B) oGALT**	**I**	**II**	**III**	**Total**	
	**I**	6	0	0	6	≤ 0.001
	**II**	3	9	0	12	
	**III**	0	3	6	9	
	**Total**	9	12	6	27	
	**C) Intestinal LNN**	**I**	**II**	**III**	**Total**	
	**I**	1	3	0	4	0.703
	**II**	1	5	2	8	
	**III**	3	7	5	15	
	**Total**	5	15	7	27	

## Discussion

A well characterized experimental model for the clinically non-apparent phase of paratuberculosis was established which fulfils the following requirements for the experimental design [18]: (1) defined host, (2) standardized infection, (3) standardized stage of disease, (4) appropriate controls.

Goats of the breed Thüringer Wald Ziege were selected as host species. This is an independent native German breed of dairy goats which has been maintained without cross-breeding since 1935 [19]. All kids came from one pedigree herd with no history of clinical paratuberculosis. Freedom from disease had been verified by the negative outcome of faecal culture of all adult goats prior to purchase of the kids and was confirmed by lack of lesions as well as negative results of faecal and tissue culture of all control animals.

For standardization of the infection special attention was paid to the strain selection and preparation of the inoculum. The isolate used for inoculation (JII-1961) is a Type II (C-) strain of MAP genetically highly similar to K10 (P. Möbius, personal communication). It was isolated from the ICV-LN of a dairy cow with paratuberculosis [20]. The inoculum was prepared from a bacterial stock which had been established after a few passages of the isolate. Aliquots of this stock are available for further experiments. The MAP dose was standardized as recommended [5] by adjusting the bacterial wet mass used for preparation of the inoculum.

Four different inoculation regimens were compared in order to optimize the model to be representative for the clinically non-apparent phase of the disease. Three animals of the group inoculated with the high dose at early age developed neurological signs which could not be attributed to MAP infection. An association between cerebrocortical necrosis and paratuberculosis has not been reported in the literature.

In addition to clinical signs, the course of the disease was monitored in vivo by four parameters (faecal shedding, specific serum antibodies against MAP, antigen-induced IFN-γ and IL-10 response), which have also been used by other investigators (reviewed by [5]), thus allowing comparison between studies. The blood parameters in particular were seen as surrogates of host-pathogen interactions in clinically healthy animals.

The experiment was terminated about one year after inoculation independent of the appearance of clinical signs. At that time, the proportion of animals that developed clinical signs characteristic of paratuberculosis was rather low and independent from group allocation. Tissue lesions and level of tissue colonization with MAP were quantified as measure for the manifestation and dissemination of the infection. The results confirmed that the majority of the inoculated goats were in the clinically non-apparent phase of paratuberculosis. The results of the in vivo parameters indicate that even the animal without signs of infection at necropsy (goat no. 39) had undergone transient infection.

Appropriate numbers of age-matched control animals of the same origin were included in the study and examined in the same manner as the inoculated goats. The animals were kept in a separate room of the same animal facility as the inoculated groups to assure similar environmental, feeding and housing conditions. They were neither exposed to nor infected by MAP as proven by the results of the examinations conducted in vivo and after necropsy. This confirms that the applied sanitary and management measures were sufficient to prevent unwanted carry-over of MAP.

Onset and time course of faecal shedding, antibody response, and antigen-specific IFN-γ response was similar to one [10] and completely different from another study in goats [6], further supporting the need for standardization of the model. In both studies the total inoculation doses were considerably higher than in the present one, and the goats were challenged with a MAP strain of caprine origin whereas our goats received an isolate of bovine origin. Differences in virulence or host adaptation of the MAP strains used for inoculation have to be taken into account. However, direct comparison of the virulence of bovine and caprine MAP isolates for goats has not been performed yet. Higher responsiveness and/or a quicker onset of the host response to C-strains of MAP in comparison to S-strains has been demonstrated in sheep, goats, cattle [16,17,21] and deer [22]. Another explanation might be a different susceptibility of different goat breeds to MAP although scientific evidence is lacking [23].

In the present experiment, IFN-γ response and antibody response started almost at the same time. This matches with findings in naturally infected goats where the onset of the IFN-γ response usually preceded the humoral response, but positive antibody titers could sometimes be seen simultaneously with, or even prior to the IFN-γ response [24]. The paradigm of a Th1 over Th2 dominancy in the early stages of MAP infection, that has been postulated for many years, is challenged by these data, which are further confirmed by the results of experimental MAP infections of sheep [25]. Considerable variation of the individual host responses was seen in the further course of the experiment which is in agreement with findings in naturally infected goats [24,26] as well as experimentally infected sheep [21,25] and cattle [16].

Transient elevation of antigen-induced IL-10 release by PBMC was observed in MAP-inoculated and control goats up to 14 wpi depending on the age at inoculation, with significantly higher levels in MAP-inoculated animals compared to age–matched controls (K1) in the early inoculated groups (V1, V2). Similar findings were reported from calves after experimental oral inoculation of mucosal scrapings from a cow with clinical paratuberculosis [27]. It can be speculated that a transient anti-inflammatory response is induced in very young goats shortly after MAP infection which is subsequently down-regulated by a strong IFN-γ response indicative of pro-inflammatory mechanisms. In a leprosy model, IFN-γ differentially modulated IL-12 and IL-10 production resulting in up-regulation of IL-12 and down-regulation of IL-10 release in response to *Mycobacterium leprae* stimulation [28].

Morphological investigation and cultivation of MAP from tissues collected at necropsy one year after inoculation were used to confirm the infection. Lesions and/or MAP were detected in 26 of the 27 goats inoculated indicating a very high infection rate. This has been reported in several other studies in goats and confirms the good reproducibility of MAP-infection using this model [10,17,29,30]. Lesions induced by the experimental infection were comparable to those in natural infection [31,32]. There were minor differences: (1) Naturally infected goats had a higher frequency of diffuse lesions in the small intestine indicating a more advanced stage of infection in the goats sampled at slaughter [31,32]. (2) JPP and ICVPP were most consistently affected in the present and other experimental studies [30,33], whereas lesions predominated in IPP, terminal ileum and ICVPP in naturally infected goats and also some experimental studies [29,31,32,34]. Lesions were seen in intestinal lymph nodes in similar frequency as in oGALT, whereas other studies report a predominance of lesions in intestinal lymph nodes [29]. These differences might be related to the sampling procedures. (4) The number of intestinal lymph nodes with caseous and calcified granulomas was markedly higher compared to naturally infected goats [31]. It remains unresolved whether virulence of the MAP strain, stage of infection or susceptibility of the breed have contributed to this. Caseous and calcified granulomas are characteristic of MAP infection in goats and not common in other ruminant species. They may allow comparative studies for other mycobacterial infections even beyond the scope of paratuberculosis.

Isolation of MAP from tissues was not possible in four of the inoculated goats, confirming findings in other experimental studies [6,7,9]. Obviously, the organism was cleared to a large extent or changed to a viable-but-non-culturable state after manifestation of infection, since all four goats shed MAP until at least 10 wpi, all mounted a specific IFN-γ response, and tissue lesions were found in three of them. Culture results were assessed semi-quantitatively, an approach utilized only in a few other published studies in goats and sheep [10,29,35]. This allowed allocation of the animals to BOB categories as a prerequisite for additional comparative analyses. In agreement with another experimental study [6] viable MAP was most often recovered from lymph nodes and to a lesser extent from intestinal mucosa and oGALT. In contrast, no difference in the proportion of positive lymph nodes and intestinal samples was obvious in naturally infected adult goats [26]. This seems to be due to the more advanced stage of the disease in these animals in comparison to the experimental studies. Viable MAP was also recovered from extra-intestinal sites, pointing to dissemination of the organisms already in the clinically non-apparent phase of the disease and confirming findings in experimentally [10] and naturally infected goats [26].

Two different doses of MAP and two different age periods for inoculation were compared in order to identify the optimal infection regimen. There was considerable inter-individual variation in faecal shedding, the in vivo-host responses, BOB and lesions at necropsy, but no significant differences between the treatment groups except for an earlier onset of the IFN-γ response and a reduced IL-10 response in the animals that received high MAP doses starting at 42 days after birth (group V4). This is not unexpected, since the modifications of the inoculation dose and the age at inoculation were only minimal in the present experiment in comparison to other studies performed in sheep and deer [22,35-37].

The readout parameters, lesion scores and BOB, used at the end of the experiment were closely matching in the intestinal tract, but markedly different in the intestinal LN. Differences between lesion scores and BOB were more frequent in goats with milder lesions where oGALT and intestinal mucosa were not uniformly affected. In the intestinal LN, histological classification did not allow conclusions about BOB. In particular, low BOB was associated with no, mild and severe lesions. These differences are not unexpected, since the lesions reflect tissue damage caused by MAP and the reaction of the host to MAP, and cultural results the ability of MAP to survive, replicate and spread. It can be speculated that MAP is more efficiently cleared from intestinal LN than from mucosa or oGALT because of tissue-specific control mechanisms. The granulomas that predominated in the intestinal LN may either allow the host to control the mycobacterial infection or MAP to persist [38].

Overall lesion and BOB categories showed agreement in the majority of goats. Only in three goats, MAP was not cultivated, but caseous and calcifying granulomas were present in intestinal lymph nodes and in three goats, lesions were considered severe, but BOB was low. Two factors might have contributed to these discrepancies: (1) the massive host immune response – seen as severe lesions - may have reduced the amount of viable MAP in the tissue and (2) organization and removal of lesions after the clearance of the pathogen take an extended period of time. Lesions and BOB categories will allow subgrouping of animals for retrospective analyses of in vivo data.

## Conclusions

A well characterized experimental animal model of paratuberculosis was established in goats. The lack of clinical signs and the finding of paucibacillary lesions in the majority of goats indicate that this experimental model targets as intended the clinically non-apparent phase of infection. Monitoring of host-pathogen interaction in vivo and post mortem findings confirmed inter-individual differences in the progress of disease and will allow subgrouping for comparative investigations. This animal model provides the basis for future studies of pathogenesis and early diagnosis of MAP infection.

## Methods

### Legislation and ethical approval

This study was carried out in strict accordance with European and National Law for the Care and Use of Animals. The protocol was approved by the Committee on the Ethics of Animal Experiments and the Protection of Animals of the State of Thuringia, Germany (Permit Number: 04-002/08). All experiments were done in containment of biosafety level 2 under supervision of the authorized institutional Agent for Animal Protection. During the entire study, every effort was made to minimize suffering.

### Animals

Thirty nine male goats kids (breed: ‘Thüringer Wald Ziege’) were included. Animals originated from a conventionally raised herd of dairy goats with no history of clinical paratuberculosis. Before purchase, all adult goats of the herd were tested once for the presence of MAP by faecal culture and were proved to be negative. Clinically healthy goat kids aged 3 to 5 days and weighing between 2.6 and 6.0 kg (4.01 ± 0.76 kg; mean ± SD) were transferred to the animal facility of the Friedrich-Loeffler-Institut in Jena.

The goats were allocated to six different groups (n = 6-7) considering weight at birth, age, sire and dam in order to prevent full siblings in the same group, get an equal distribution of the offspring of one sire over the groups and adjust the mean body weight and the mean age of the groups. Throughout the entire study, animals of the different groups were kept in separate rooms but under equal housing and feeding conditions, and in accordance with international guidelines for animal welfare [39,40].

Feeding was adjusted to the age-dependent nutritional needs of the animals. The kids received goat milk from the herd of origin up to day 8 after birth, then commercial milk replacer for goat kids (Denkamilk capritop, Denkavit, Warendorf, Germany) up to the age of 10 weeks. Water, mineral blocks without copper, containing 37 % sodium, 1.1 % calcium, 0.6 % magnesium and trace elements (Mineralleckstein ohne Kupfer, esco – european salt company, Hannover, Germany) and hay were supplied *ad libitum* during the whole course of the experiment. Small amounts of pelleted concentrates were offered already during milk feeding. After weaning protein rich pelleted concentrates for goats (Alleinfuttermittel für Ziegenmastlämmer and Milchleistungsfutter II, both LHG Landhandelsgesellschaft, Schmölln, Germany) containing vitamin A, D3 and E were fed. The ration of concentrates was gradually increased up to 500 grams per day. None of the given feed contained antibiotics. The animals were castrated at the age of 12 weeks. During the whole course of the experiment the animals were neither treated with systemic antibiotics nor immunosuppressive drugs.

### Study design

Four of the six groups were challenged with MAP (V1 – V4; n = 6-7), two groups served as controls (K1, K2; n = 6, Table 6). With respect to the suggested guidelines [5,23], four different inoculation regimens were compared. Treatment of the groups differed as follows: Oral inoculation with MAP started 3–5 days post natum (dpn) for two early inoculated groups (V1, V2) and at 42 dpn for two late inoculated groups (V3, V4). Inoculation was performed ten times every two to three days. One group each of the early and the late inoculated goats received 10 mg bacterial wet mass (bwm, V1, V3) per dose, the others received 20 mg bwm of MAP per dose (V2, V4). The age matched controls were sham-inoculated at the same time (K1 starting at 3–5 dpn, K2 starting at 42 dpn, Table 6).

**Table 6:** **Study design: group allocation, time point and dose of inoculation and time point of necropsy**

**Start of inoculation period**	**3-5 dpn**			**41-43 dpn**		
**Group**	**K1**	**V1**	**V2**	**K2**	**V3**	**V4**
Number of animals (n)	6	7	7	6	6	7
Inoculation dose per day (mg bwm)	-	10	20	-	10	20
Average bacterial counts/mL	-	2.08 ± 0.57x10^7^		-	1.62 ± 0.73x10^7^	
Average bacterial counts/inoculum	-	2.08x10^8^	4.16x10^8^	-	1.62x10^8^	3.24x10^8^
Total inoculation dose	-	2.08x10^9^	4.16x10^9^	-	1.62x10^9^	3.24x10^9^
Time of necropsy (wpi)	48^a)^	48	49^b)^	46	47	48^c)^

dpn, days post natum; bwm, bacterial wet mass; wpi, weeks post infection; ^a)^one animal was necropsied at 39 wpi; ^b)^one animal each died at 37 and 39 wpi, one animal each was necropsied at 37 and 38 wpi; ^c)^one animal was necropsied at 35 wpi.

**Figure 7:**
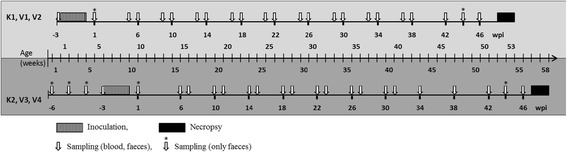
**Schematic representation of the time course of the experiment.** Challenge time and dose, animal number: V1 – 3–5 dpn, 10 mg bwm, n = 7; V2 – 3–5 dpn, 20 mg bwm, n = 7; V3 – 42 dpn, 10 mg bwm, n = 6; V4 – 42 dpn, 20 mg bwm, n = 7. Controls: K1 – 3–5 dpn, n = 6; K2 – 42 dpn, n = 6.

Each goat underwent daily clinical examination from the beginning of the experiment until necropsy. Parameters to follow the course of infection were faecal shedding of MAP, antibody response in serum and specific IFN-γ and IL-10 responses of peripheral blood mononuclear cells (PBMC). Individual blood and faecal samples were collected to examine humoral immune response and faecal shedding of MAP before the first inoculation and in regular intervals after inoculation. The amount of blood that could be collected from very young goat kids was limited because of animal welfare reasons, therefore, testing of the cellular immune response started only 5–6 weeks post inoculation (wpi) and continued until necropsy (Figure 7).

As scheduled, goats were euthanized and necropsied about 12 months after the last inoculation. A few animals died or had to be necropsied earlier for animal welfare reasons (Table 6). Gross and histologic lesions, amount of MAP in lesions detected by immunohistochemistry, and bacterial organ burden were recorded. Emphasis was laid on elaboration of detailed qualitative and semi-quantitative scoring systems for bacteriological and pathomorphological parameters.

### Preparation of bacteria used for inoculation

A low passage MAP field isolate from the ICV-LN of a cow (JII-1961) was propagated in Middlebrook 7H9 broth (MB, Becton Dickinson, Heidelberg, Germany) containing glycerine (Merck, Darmstadt, Germany), OADC (Becton Dickinson, Heidelberg, Germany) and Mycobactin J (Allied Monitor, Fayette, MO, US) at 37 ± 2 °C. No antibiotics were added. After centrifugation, the bacterial pellet was recovered. The pellets from different parallel culture batches originating from the same pre-culture were re-suspended in small volumes of culture medium, pooled, and dispensed to form the bacterial inoculum stocks. After centrifugation of the stocks, the bacterial wet mass (bwm) of each pellet was determined. The stocks were stored at 5 ± 3 °C for a maximum of 9 weeks. One stock was used per inoculation day. Two to three days before inoculation the pellet was re-suspended to a concentration of 1 mg bwm/mL using PBS. This batch suspension was incubated at 37 ± 2 °C until the day of inoculation. Then, either 10 mL (groups V1 and V3) or 20 mL (groups V2 and V4) of the suspension were dispensed into separate tubes to form the inoculum for each individual animal. Bacterial counts of the respective batch suspension were determined on plates of Middlebrook 7H10 agar (Becton Dickinson, Heidelberg, Germany) containing OADC, Mycobactin J and Amphotericin B (AppliChem, Darmstadt, Germany). Serial dilutions from 10^−1^ to 10^−9^ were performed in MB and 100 μl of each dilution was plated on the agar plates. Plates were incubated at 37 °C for six weeks. Colony numbers were counted from the three highest dilutions showing visible colonies. Bacterial counts were expressed as the mean of the colony numbers corrected for the dilution. The bacterial inoculum amounted to 1.62 – 4.16 × 10^8^ cfu/dose (Table 6). For further characterization of the batch suspensions, acid-fast bacilli (AFB) were confirmed by Ziehl-Neelsen staining, MAP was confirmed by IS*900* PCR using primers according to Englund et al. [41]. Freedom from contaminating bacteria was proved by inoculation on blood agar plates.

### Oral administration

Each individual MAP dose was suspended in 50 mL of pre-warmed milk replacer in a baby bottle. The goats were bottle-fed with the inoculum prior to regular morning feeding. Control animals received the same amount of pure milk replacer.

### Faecal culture

Three gram of faeces were placed into 30 mL of 0.75% HPC. The solution was mixed vigorously, allowed to settle for five min and the supernatant transferred to a fresh vial. The samples were agitated on a shaker for 30 min and then incubated in upright position for 48 hours at room temperature (RT) in the dark. The supernatants were discarded and 200 μL of the pellet were transferred on each of four slopes of Herrold’s Egg Yolk Medium with Mycobactin J and Amphotericin, Nalidixic acid and Vancomycin (ANV, HEYM, Becton Dickinson, Heidelberg, Germany). The cultures were incubated up to 6 months at 37 ± 2 °C and checked every 2 weeks for contamination and occurrence of visible colonies. As soon as colonies became visible, colony counts were estimated semi-quantitatively by a colony score (CS) from 1 to 5, with 1 = 0 – 10; 2 = 11 – 20; 3 = 21 – 50; 4 = 51 – 100 individual colonies per slope; 5 = bacterial lawn, and the week of appearance (WA) was recorded. To correct for the time until colonies became visible, a growth index (GI) allowing a numerical estimation of the MAP concentration in the samples was calculated by the formula: GI = CS × 100/WA. The mean GI of the four corresponding slopes was determined.

### Serum preparation and antibody detection

Blood without anti-coagulants was kept for 2–3 hours at RT, centrifuged at 2000 × g for 20 min, the serum recovered, aliquoted and stored at −20 °C until use. MAP specific antibodies were detected with the ID Screen Paratuberculosis Indirect ELISA (ID Vet, Montpellier, France) according to the instructions of the manufacturer. The antibody response is demonstrated by the sample-to-positive ratio (S/P%) as recommended by the manufacturer.

### Preparation of PBMC

Twenty mL blood containing 75 units per mL sodium-heparin (SIGMA Taufkirchen, Germany) was layered over 20 mL lymphocyte separation medium (PAA, Pasching, Austria) and centrifuged at 1500 × g for 30 min at RT. The PBMC were removed, washed three times with Hank’s buffer without calcium and magnesium, re-suspended in RPMI 1640 medium with glutamine, 10% foetal calf serum (FCS), 10 mM HEPES and 1% Penicillin/Streptomycin (all Biochrom, Berlin, Germany) and adjusted to 2 x 10^6^ PBMC per mL.

### Stimulation assay

PBMC were transferred to 96-well round bottom culture plates (NUNC, Roskilde, Denmark) to a final cell number of 2 x 10^5^ PBMC per well. Cells were stimulated in triplicate with Concanavalin A (Con A, 20 μg/mL, SIGMA, Taufkirchen, Germany) or Johnin purified protein derivative (jPPD, 4 μg/mL, kindly provided by D. Bakker, CVI-WUR, Lelystad, The Netherlands) at 37 ± 2 °C, 5% CO_2_. Non-stimulated cells served as controls. Cell supernatants were collected after 24 hours of stimulation for measurement of IFN-γ and after 64 hours for IL-10.

### IFN-γ ELISA

IFN-γ was measured with an in-house capture ELISA using monoclonal antibodies against bovine IFN-γ (capture antibody: clone CC330, AbD Serotec, Kidlington, UK; detection antibody: clone CC302-Biotin, AbD Serotec), HRP-labelled biotin as conjugate (AbD Serotec) and 3,3′,5,5′-tetramethylbenzidine (TMB) as substrate (SIGMA, Taufkirchen, Germany). IFN-γ concentration was determined relative to a dilution series of recombinant bovine IFN-γ (AbD Serotec) from 9.167-0.013 ng/mL.

### IL-10 ELISA

IL-10 was measured with an in-house capture ELISA using monoclonal antibodies against bovine IL-10 (capture antibody: clone CC318, AbD Serotec; detection antibody: clone CC320-Biotin, AbD Serotec), HRP-labelled biotin and TMB as described for the IFN-γ ELISA. IL-10 concentration was determined relative to a dilution series of recombinant bovine IL-10 (kindly provided by G. Entrican and S. Wattegedera, Moredun Research Institute, Penicuik, Scotland, UK) from 30–0.041 U/mL.

The validity of both, the IFN-γ and the IL-10 ELISA for caprine samples was determined in preceding stimulation experiments using PBMC of healthy adult goats (data not shown).

### Necropsy and collection of tissue samples

Goats inoculated with MAP were necropsied between 47 and 49 wpi and controls 46 and 48 wpi. Special sampling was done to obtain intestinal samples of high quality. At necropsy, the sites of intestine to be collected for histology were selected while the animals were under deep anesthesia. For this, they were sedated with Xylazin at a dose of 0.25 mg/kg body weight (BW) IM (Rompun® 2%, Bayer, Leverkusen, Germany) and anesthetized with Ketamin hydrochloride at 2.5 mg/kg BW IV (Ketamin 10%, Intervet, Unterschleißheim, Germany) and Diazepam at 0.5 mg/kg BW IV (Faustan®, AWD, Radebeul, Germany) [42]. The intestine was exposed, segments of about 5 cm were ligated and filled with 4% neutral buffered formalin (NBF) in the duodenum, four sites of jejunum (3 m apart), one jejunal Peyer’s patch (JPP) from the proximal and from the distal jejunum and three sites (terminal ileum, 50 cm and 150 cm proximal to the ileocaecal valve (ICV)) of ileal Peyer’s patch (IPP). Then, goats were euthanized with 20 mL of pentobarbital IV (Release®, WDT, Garbsen, Germany), the intestine removed *in toto*, detached from the mesentery and spread full length on a table. In addition to the sites mentioned above, samples were collected from caecum, organized lymphoid tissue in the colon next to the ileocaecal valve (ICVPP), at the end of the proximal colon (PCPP) and in the rectum, central flexure of the colon and descending colon. JPP, IPP, ICVPP and PCPP are referred to as organized lymphoid tissue in the intestinal wall (oGALT). Samples for histology were opened, pinned flat on styrofoam and fixed in NBF. Samples were also collected at the respective sites for bacterial organ culture. The remaining intestine was opened, ingesta and mucosa examined and macroscopic lesions documented.

All intestinal lymph nodes were examined and samples collected from proximal, mid (only bacterial organ culture) and distal mesenteric lymph nodes (M-LN), ileocolic lymph nodes (ICV-LN) and colonic lymph nodes (Co-LN, only histology). Then a complete necropsy was performed and representative tissue samples were collected from tonsils, retropharyngeal LN, spleen, kidney, liver, hepatic LN for cultural isolation and histology, diaphragm, gluteal muscle, and superficial cervical LN (only for cultural isolation), and from thymus, lung, heart, aorta, pancreas, adrenal, rumen, abomasum, bone marrow, and superficial inguinal LN (only for histology).

### Histology and immunohistochemistry (IHC)

Tissue samples were embedded in paraffin and lesions examined in hematoxylin and eosin (HE)-stained paraffin sections. Severity of lesions was graded from + to +++, with + (mild), ++ (moderate) and +++ (severe), distribution of lesions as focal (f) – up to 3 distinct granulomatous infiltrates per section; multifocal (mf) – more than 3 distinct granulomatous infiltrates per section and diffuse (d) – infiltrates throughout the section. In the intestinal lymph nodes, granulomatous infiltrates and distinct granulomas with central necrosis and calcification were seen. In oGALT the following types of lesions were distinguished: A – small foci of epitheloid cells and multinucleated giant cells without changes of the tissue architecture; B – extensive granulomatous infiltrates with many lymphocytes; C – small foci of granulomatous infiltrate embedded in numerous lymphocytes and plasma cells and atrophy of the organized lymphoid tissue; D – multifocal to diffuse granulomatous infiltrates with numerous epitheloid cells.

Paraffin sections of all intestinal sites, intestinal lymph nodes, liver, tonsil, hepatic LN, superficial inguinal LN and tissues with granulomatous lesions were examined for MAP by the indirect immunoperoxidase method. Polyclonal rabbit anti-MAP serum (Dako, Glostrup, Denmark) was used as primary antibody and peroxidase-conjugated goat anti-rabbit IgG as secondary antibody (Dianova, Hamburg, Germany). Sections were pretreated with trypsin (0.1%, 37 °C, 20 min) for antigen retrieval. 3-amino-9-ethyl carbazole (AEC) was used as chromogen. Sections were counter stained with hematoxylin. As positive control, a slide from an experimentally inoculated goat in which mycobacteria had been detected by Ziehl-Neelsen staining and from which MAP had been isolated by culture was included. As negative control, a consecutive section of the positive control was incubated with a polyclonal antiserum directed against non-related bacteria (*Brachyspira hyodysenteriae*) instead of the polyclonal rabbit anti-MAP serum. For each section which had areas of necrosis, a consecutive section was incubated with the antiserum against non-related bacteria as negative control. The number of mycobacteria per section was graded using a scoring system adapted from previous studies as none (less than two labeled bacteria per section), paucibacillary (up to 50% of macrophages contain MAP, on average 1–10 MAP per macrophage, few areas in necrotic centers of granulomas) and multibacillary (most macrophages contain MAP, on average >10 MAP per macrophage or uncountable, extensive areas in necrotic centers of granulomas) [43].

Macroscopic lesions, histological findings and immunohistochemical detection of MAP were summarized as lesion categories for the intestinal lymph nodes, oGALT, small intestine and as overall lesions for the individual animal to be able to correlate the morphological findings with cultural isolation of MAP from organs, faecal shedding and immune reactions. The different sites were discriminated, because severity of lesions varied within individual goats. The criteria are listed in Table 7.

**Table 7:** **Assignment of lesion categories for mesenteric and ileocecal LNN, oGALT and small intestine and in summary**

**Lesion category**	**mesenteric and ileocecal LNN**	**oGALT**	**small intestine**	**summary**
I	no lesions (macroscopic, histological), no MAP	no lesions (macroscopic, histological), no MAP	no lesions (macroscopic, histological), no MAP	all 3 sites (LNN, oGALT and small intestine) are category I
II	predominantly small circumscribed lesions, lesions in 1–2 out of the 3 LNN examined, paucibacillary	lesions in up to 3 sites of oGALT, f/mf, paucibacillary	lesions in up to 2 sites of small intestine, f/mf, paucibacillary	at least 2 of the 3 sites (LNN, oGALT, small intestine) are category II **or** 1 site is category II, 2 are category I **or** 1 site each is category I, category II and category III
III	predominantly extensive lesions, lesions in all 3 LN, pauci-/multibacillary	lesions in >3 sites of oGALT, mf, pauci-/ multibacillary	lesions in >2 sites of intestine, mf, pauci-/ multibacillary	at least 2 of the 3 sites (LNN, oGALT, small intestine) are category III **or** 1 site is category III and the others are category I

LNN lymph nodes, oGALT gut-associated lymphoid tissue, f focal, mf multifocal.

### Tissue culture

Fat and connecting tissue was removed from lymph nodes and other tissue. Intestine was opened and ingesta removed. One gram of sample was cut from different locations, minced with scissors and transferred into a plastic bag containing 7 mL 0.9% HPC. The samples were homogenized in a stomacher for 6 min, transferred to a 50 mL tube and agitated on a shaker at 200 rpm for 10 min at RT. Afterwards they were incubated in upright position for 24 hours at RT in the dark. After centrifugation at 1880 × g for 20 min at RT, supernatants were discarded and the pellet re-suspended with 1 mL of sterile phosphate buffered saline (pH 7.2). 200 μL of the pellet were transferred on each of four slopes of HEYM (Becton Dickinson, Heidelberg, Germany). The cultures were further treated and GI was calculated as described above.

The bacterial organ burden (BOB) was calculated for the samples from intestine, oGALT and intestinal lymph nodes separately and in summary by dividing the sum of the GI’s of the respective tissues by the number of tissues involved. BOB categories were designated as follows: category I – no growth, category II - BOB ≤ 15, category III - BOB > 15.

### Statistics

Response intensities of inoculation groups were analysed with the Mann–Whitney U-test, frequencies of categories of parameters were compared using Pearson’s χ^2^-test. The level of significance for all statistical methods applied was P ≤ 0.05. Results were displayed as ‘Box and Whisker’ plots. Outlier values are 1.5-3 times of the length of a box away from the median and extreme values are further away than three times of the length of the box.

## Abbreviations

AFB: Acid fast bacilli
BOB: Bacterial organ burden
bwm: Bacterial wet mass
dpn: Days post natum
oGALT: Organized lymphoid tissue in the intestinal wall
GI: Growth index
ICV: Ileocaecal valve
IFN-γ: Interferon-γ
IL-10: Interleukin-10
IPP: Ileal Peyer’s patch
JPP: Jejunal Peyer’s patch
jPPD: Johnin purified protein derivative
LN: Lymph node
MAP: Mycobacterium avium subsp. paratuberculosis
mpi: Months post infection
PBMC: Peripheral blood mononuclear cells
wpi: Weeks post infection
